# A Therapeutical Arrangement of the Materia Medica, or the Materia Medica Arranged upon Physiological Principles, and in the Order of the General Practical Value Which Remedial Agents Hold under Their Several Denominations, and in Conformity with the Physiological Doctrines Set Forth in the Medical and Physiological Commentaries

**Published:** 1842-07

**Authors:** 


Art. I.—*A Therapeutical Arrangement of the Materia Medica, or the Materia Medica arranged upon Physiological Principles, and in the order of the General Practical Value which remedial agents hold under their several denominations, and in conformity with the physiological doctrines set forth in the Medical and Physiological Commentaries.* By Martyn Paine, m.d. a.m., Author of the Commentaries, &c.—*New York,* 1842. Sm. 8vo, pp. 271.

Notwithstanding the obligations we lie under to the author of the small volume whose very characteristic title we have just transcribed, we are compelled, by a sense of duty, to state that it is not a very superior production. We cannot but regret the necessity of making this avowal, seeing that Dr. Paine hath honoured us of late, and we have bought golden opinions from all sorts of people, through his instrumentality. By means of various communications in the American journals, and sundry separate publications, having reference to this Review, its editor and contributors, which have been carefully transmitted by post to all the most eminent men in the profession in Great Britain, and even on the continent; and, lastly, by the kind dedication of the present volume "to the forbearing consideration of Dr. John Forbes," and to his "justice honour," he has not merely carried our fame into new regions, given our names a fresh impulse—

Virum volitare per ora, but has actually ministered to our gratification on a point generally considered as touching all men most nearly, viz. the improvement of our exchequer. It is a fact which Dr. Paine can any day verify, by a reference to our New York publishers, that in the quarter immediately succeeding his great advertisement of this journal, the demand for it in America was increased one twelfth! But we are sure that Dr. Paine, with that liberality of sentiment, cool judgment, and ready credence of the assertions of men of honour, so characteristic of him, will not only forgive us for remaining unseduced by his moving blandishments, but will commend us (may we hope in another pamphlet?) once more to the medical world as the greatest of journalists, and the most impartial of critics.

After this long and exculpatory preface, it may be expected that we are going to be very severe upon Dr. Paine's volume. In one respect, this is the case, as *we* feel it to be a severe judgment to be obliged to say of so good a friend that he has written a book that is not first-rate.

The title-page, which we have copied nearly at full length, will give the reader a general idea of the nature of this volume. The "arrangement" adopted by the author is somewhat different from that of preceding authors, but not so much so as to be very noticeable by his readers. The following are the names of his "Classes," and of the "Orders" contained in the first class:

"Classes. i. Antiphlogistics; ii. Permanent Tonics; iii. Diffusible Stimulants; iv. Cerebro-Spinants, or Nervous Agents; v. Astringents; vi. Uterine Agents; vii. Urinary Agents; viii. Anthelmintics; ix. Errhines; x. Chemical Agents; xi. Diet and Regimen, in a general sense.

Orders. Class I.—*Antiphlogistics.* 1, Bloodletting; 2, Cathartics; 3, Emetics; 4, Alteratives; 5, Expectorants; 6, Direct Sedatives; 7, Diuretics; 8, Cutaneous and other applications; 9, Low diet and rest. (*Negative.*)

One feature of the present volume leads us to entertain a very exalted opinion of the intelligence and industry of the medical pupils in America. Dr. Paine, of course, knows their capacity and temper, and has too much sense to draw up for their especial use a book which they could not, or would not, understand. It is, however, certain that, in the particular referred to, Dr. Paine has gone much beyond the reach, if not of the powers, certainly of the will of the medical pupils in this country. We can truly add, that he has gone beyond *our* reach also; but we wish to lay little stress on this circumstance, as, on other occasions, we have acknowledged our feeble powers to be inadequate to follow the vigorous wing of Dr. Paine. Perhaps we are mistaken in thinking that the author's good nature has led him to place too much confidence in the zeal and industry of his readers, in believing that they will patiently work out from all quarters of his book the substantial materials of the numerous formulæ, of which he gives the mere symbols under the head of the individual articles of the materia medica. Of the accuracy of this opinion of ours—and we cannot but have some mistrust of any opinion at variance with that of Dr. Paine—we leave our readers to judge from the following extracts, which we take from the beginning of the volume, and which are fair specimens of the work. In order that we may place our readers in the same position as ourselves, in regard to the understanding of the extracts, we premise his "Instructions" as to the manner in which the symbolic formulæ are to be understood and elaborated. We hope none of our readers will be ill-natured enough to exclaim, with Dangle, in the Critic, "Egad! I think the interpreter is the hardest to be understood of the two!"

"For the sake of brevity, and to impress the memory, characters or symbols are frequently employed to indicate the combinations of remedies. The initial letters following the usual ℞ stand for the article with which the formula is connected. If a figure follow next, it stands for the article so numbered in that group. If an article be derived from another class, the word *class* is introduced, and the number following indicates which class. Then follows a number referring to the intended article in that class; or, if the class have orders or subdivisions, the word *order* or *sub.* with the number or letter indicative of which *order* or *subdivision,* precedes the number which stands for the remedy. If several numbers succeed each other uninterruptedly, after the announcement of the class, etc., they all refer to remedies under the same group."

"Jalap. The best formulæ are the following:—℞. J. 1. ℞. J. 1 and order 3, 1. ℞. J. 1, and order 4 A. 18 or 19. ℞. J. 11, ℞. J. 11, 12. ℞. J. 1, 12. ℞ J. order 4 A. 18 or 19, class 3. 12.[a] ℞. J. 23. ℞. J. infus. 6.[a] ℞. J. infus. 12.[c] ℞. J. infus. class 8. 2."

"Aloes. See Calomel and Blue Pill for several formulæ; also, ℞. a, 1. order 4. A. 18, class 4, order 1, 13, 16. ℞. a, 12, 52. ℞. a. 16,[b] 17. ℞. a. 16,[b] 17, 18, 31, order 4. A. 13. ℞. a. 1 or 2, class 4, order 1, 16. ℞. a. 1, 16,[b] 17, class 2, 15. ℞. a. 17, 12.[e] class 3, 14.[b] ℞. a. 12, 21, class 2, 9. ℞. a. order 4. A. 1, 13, Sub. 5, 1. ℞. a. 18, class 2, 43. ℞. a. 16.[a] 3,[b] 31, 17. class 4, order 1, 16."

"Rhubarb. ℞. R, 14. ℞. R. 1. ℞. R. 1. 3. ℞. R. 7. ℞. R. 6. 52. ℞ R. 6, 17. ℞. R. 22, class 3, 9.[b] ℞. R. 7. 14. ℞. R. 14, class 3, 11.[c] ℞. R. class 3, 12,[c] class 4. order 1, 11. class 10, 1."

